# A Serotoninomic Framework for Reproductive and Integrative Toxicology: Molecular, Neurochemical, and Behavioural Perspectives on Permethrin Exposure

**DOI:** 10.3390/toxics14050365

**Published:** 2026-04-24

**Authors:** Francisco Jiménez-Trejo, Liliana Carmona-Aparicio, Elvia Coballase-Urrutia, Katia L. Jiménez-García, Cristian Arriaga-Canon, Luis A. Herrera

**Affiliations:** 1 Laboratorio de Morfología Celular y Tisular, Instituto Nacional de Pediatría, Mexico City 04530, Mexico; trejofjj@gmail.com; 2 Laboratorio de Farmacología, Instituto Nacional de Pediatría, Mexico City 04530, Mexico; c_apariccio@yahoo.com.mx; 3 Departamento Psiquiatria, Facultad de Medicina, Universidad Nacional Autónoma de México, Mexico City 04510, Mexico; katiajimenezz03@gmail.com; 4 Laboratorio de Innovación y Medicina de Precisión, Instituto Nacional de Medicina Genómica, Mexico City 14610, Mexico; cgarriaga@inmegen.gob.mx; 5 Escuela de Ciencias Médicas y de la Salud, Tecnológico de Monterrey, Mexico City 14380, Mexico; lherreram@tec.mx; 6 Instituto Nacional de Ciencias Médicas y Nutrición Salvador Zubirán, Mexico City 14080, Mexico

**Keywords:** serotoninomics, permethrin toxicity, reproductive neuroendocrinology, gonadal serotonergic signaling, public health, reproductive agency

## Abstract

Serotoninomics, a nascent emerging discipline within the field of omics, provides a transdisciplinary framework for understanding reproductive toxicology via serotonergic signalling. This research investigates the neuroendocrine effects of permethrin, a commonly used pyrethroid insecticide often considered to pose a low risk to humans, and positions it as a model compound for evaluating reproductive susceptibility beyond conventional endocrine endpoints. It is hypothesized that serotonin, traditionally examined in neuropsychiatric contexts, plays an essential role in gonadal function, hormonal regulation, and emotional resilience. Although permethrins are generally regarded as safe, acute exposure may subtly interfere with serotonergic pathways, potentially resulting in molecular, biochemical, behavioural, and reproductive alterations. These effects could extend beyond immediate exposure, including during gestation, considering permethrins’ ability to cross the placental barrier and influence foetal development. By synthesizing evidence across molecular, organismal, and environmental domains, we advocate for a serotonergic approach to facilitate a more comprehensive assessment of risk and resilience. We emphasize the importance of fostering a transdisciplinary dialogue to redefine reproductive health through the perspectives of serotonergic vulnerability and systemic resilience.

## 1. Introduction

We introduce a serotoninomic framework to study neurochemical and neuroendocrine disruption in reproductive health, using permethrin (PERM), a commonly used insecticide in human populations, as a model compound. This method integrates molecular, biochemical, behavioural, and environmental aspects of toxicology to understand how serotonergic pathways may influence reproductive vulnerability following permethrin exposure.

PERM is often regarded as a low-risk insecticide in conventional toxicological assessments. However, emerging evidence indicates that it may interfere with serotonergic signalling, especially with chronic exposure, without reducing the importance of acute and low-dose exposures [1,2,3]. Animal studies have demonstrated reproductive and developmental toxicity at higher doses, including neuroendocrine alterations and foetal loss, though further research is needed [4,5,6]. Recent studies also link Extracellular Signal-Regulated Kinase and Cyclooxygenase-2 pathways to testicular dysfunction and oxidative stress [2]. These disruptions, although subtle, could greatly affect emotional resilience and hormonal balance.

By integrating biochemical evidence with reproductive outcomes, we aim to discover new markers of risk and resilience and develop interpretive frameworks that go beyond reductionist toxicology. This work is more than a molecular or biochemical study; it requires interdisciplinary dialogue where neurochemistry, reproductive biology, and environmental health collaborate to redefine how we assess and address endocrine and neurological disruption, not only from a scientific perspective but also by considering ethical and emotional aspects.

## 2. Background and Rationale: Contextualizing Serotoninomics Within Reproductive Toxicology

Serotoninomics is an emerging omics discipline that aims to map, interpret, and influence the diverse functions of serotonin across various physiological systems. Initially introduced by our research team [7], it serves as an integrated conceptual framework that consolidates and personalizes all research on serotonin and its related systems, including biosynthesis, metabolism, receptor pharmacology, transport mechanisms, and functional roles within biological systems. Rather than constituting a single analytical platform, this omics science covers the full range of laboratory techniques and methodological tools historically and currently used in serotonin research: from classical biochemical methods such as HPLC-based quantification of 5-HT and its metabolite 5-HIAA, radioimmunoassay, and enzyme activity assessments to histochemical and immunofluorescence techniques for cellular localization, as well as emerging molecular strategies including gene expression analysis, receptor binding studies, pharmacological modulation with agonists and antagonists, and neuroimaging. Importantly, this approach is not equivalent to a systems-biology perspective on the serotonin pathway; instead, it promotes an integrated approach—combining molecular, cellular, behavioural, and clinical aspects to fully understand the biological complexity of this indolamine in health and disease. Similar to genomics and metabolomics, it offers a transdisciplinary framework that unites molecular biology, neurochemistry, behavioural science, and environmental health [8,9]. This methodology goes beyond a narrow focus on serotonin alone; it represents a methodological and epistemological advancement, encouraging the development of new tools, biomarkers, and interpretive models that recognize serotonin’s systemic influence [10].

Our group has actively contributed to the development of Serotoninomics, recognizing its global importance in addressing complex health issues, including its role in neuropsychiatric disorders and the kynurenine/anthranilic acid pathway [11], longevity and geriatric health [12], and broader perspectives for serotonin research in the current decade (9). This approach is particularly vital in reproductive toxicology. Serotonin (5-HT) is found in gonadal tissues, regulates hormonal feedback loops, and influences sexual behavior and emotional bonding [13,14].

Recent studies have identified serotonergic components in sperm cells, highlighting their role in post-ejaculatory fertilization processes, including capacitation, the acrosome reaction, and early signalling at the oocyte interface [15].

Despite these findings, serotonin remains largely overlooked, particularly within the realm of reproductive toxicology, where the emphasis persists on steroid hormones and conventional endocrine disruptors. Serotoninomics challenges this hierarchy by positioning serotonin as both a target and a mediator of reproductive vulnerability. PERM serves as an illustrative model compound; it is widely utilized and generally regarded as safe following acute exposure. However, its potential to disrupt serotonergic signalling, especially during gestational and pubertal periods, remains inadequately understood [16].

Applying serotoninomic principles, we aim to investigate not only molecular disruptions but also behavioural and emotional outcomes using integrative methods, including receptor profiling, gene expression analysis, and behavioural testing [17,18]. This approach is both scientific and epistemological. It challenges reductionist toxicology and promotes a broader, more compassionate science: one that redefines serotonin as a connection between biology, emotion, and resilience.

## 3. Mechanisms of Serotonergic Impacts: Pathways, Receptors, and Neuroendocrine Crosstalk

### 3.1. Serotonin Biosynthesis, Localization, and Metabolism

L-tryptophan (L-Trp) is an essential amino acid and the main precursor for serotonin (5-HT) production in both the brain and peripheral tissues. In the central nervous system, L-Trp is hydroxylated in serotonergic neurons by tryptophan hydroxylase 2 (TPH2; EC 1.14.16.4), while TPH1 is mainly found in peripheral tissues such as the gastrointestinal tract, gonads, and other organs. Both enzymes are encoded by separate genes and serve as rate-limiting steps in serotonin biosynthesis in central and peripheral areas [7]. The intermediate, 5-hydroxytryptophan, is then decarboxylated to produce 5-HT. Serotonin breakdown mainly occurs through monoamine oxidases A and B (MAOA, MAOB), which oxidatively deaminate 5-HT to form its major metabolite, 5-hydroxyindoleacetic acid (5-HIAA). Intracellular storage and vesicular release are controlled by vesicular monoamine transporters VMAT1 and VMAT2, while membrane reuptake is managed by the serotonin transporter (SERT; 5-HTT), which terminates serotonergic signalling at the synapse and in peripheral tissues [7].

Beyond 5-HT and 5-HIAA, the serotonin pathway produces active intermediates whose reproductive relevance is often underestimated in toxicology. Fully understanding permethrin’s impact on reproductive tissues requires considering this complete metabolic cascade [9]. The initial phase of serotonin catabolism involves MAO-mediated oxidative deamination, which converts 5-HT to 5-hydroxyindoleacetaldehyde (5-HIAL)—a reactive aldehyde intermediate with established cytotoxic and pro-oxidant properties. Subsequently, 5-HIAL is directed along one of two competing enzymatic pathways: oxidation by aldehyde dehydrogenase (ALDH) produces 5-HIAA, whereas reduction by aldehyde reductase (ALR) or alcohol dehydrogenase (ADH) results in 5-hydroxytryptophol (5-HTOL), which is later eliminated as glucuronide or sulphate conjugates. This reductive pathway exhibits metabolic sensitivity to fluctuations in NADH/NAD^+^ ratios—a ratio that can be influenced by oxidative stress, such as that induced by permethrin exposure. Consequently, the accumulation of 5-HTOL serves as a potential biomarker of serotonergic disruption under conditions of redox imbalance in gonadal tissues [19,20].

The serotonin pathway branches into an anabolic branch that affects reproduction. In pineal and gonadal tissues, 5-HT is acetylated by AANAT to form NAS, which is then methylated by HIOMT/ASMT to produce melatonin—the final product of the serotonergic cascade [20,21]. NAS is also a biologically independent molecule with neuroprotective and pro-fertility effects, including activation of TrkB (BDNF) receptors in a BDNF-independent, circadian manner—a property unique to NAS, not to serotonin or melatonin [22,23].

#### Reproductive Roles of Downstream Serotonin Metabolites in Gonadal Tissues

The biological significance of these metabolites in reproductive organs is substantial and specific. Melatonin, synthesized locally in gonadal tissues from the serotonin precursor cascade, exerts well-documented pleiotropic effects on both male and female reproduction. In the testis, melatonin acts via MT1 and MT2 receptors expressed in Leydig cells, Sertoli cells, and spermatogonial stem cells to: (i) regulate testosterone secretion through Leydig cell signaling; (ii) modulate Sertoli cell glycolytic metabolism specifically increasing glucose consumption and protecting lactate-dependent germ cell nutrition, thereby supporting spermatogenesis; and (iii) protect spermatogonial stem cells from oxidative damage induced by chemotherapy, radiation, and environmental toxicants [24,25,26]. Melatonin also regulates sperm quality throughout the reproductive tract: within the epididymis, it modulates sperm maturation and epididymal epithelial secretory activity; in seminal plasma, it protects spermatozoa from oxidative damage and premature capacitation; and in the female reproductive tract, low melatonin concentrations promote capacitation via MT2 receptors, while high concentrations exert a decapacitating effect [27]. In the ovary, melatonin scavenges reactive oxygen species generated during the peri-ovulatory phase, contributes to oocyte maturation, enhances embryo development, and promotes luteinization of granulosa cells [28,29].

5-HIAA itself, while classically regarded as an inert urinary biomarker, has been shown to increase specifically in brain regions following permethrin exposure in rodents—constituting direct evidence that pyrethroid-induced serotonergic disruption produces measurable downstream metabolic consequences detectable in the urinary metabolome [30]. In reproductive tissues, altered 5-HIAA/5-HT ratios reflect serotonin turnover dynamics that can signal states of oxidative stress and neuro-transmitter depletion in gonadal compartments.

5-HTOL, by contrast, is rarely quantified in reproductive toxicology studies but represents a metabolic window into redox status within spermatogenic cells, where NADH generation is tightly linked to mitochondrial function and ATP-dependent flagellar motility. Shifts in the ALDH/ALR balance toward 5-HTOL production signal mitochondrial dysfunction a mechanism directly relevant to PERM’s documented interference with sperm bioenergetics [31].

From a Serotoninomic toxicology perspective, permethrin’s disruption of serotonergic pathways cannot therefore be reduced to alterations in 5-HT or 5-HIAA alone. It must be understood as a cascade effect that propagates through each metabolic node: from altered TPH1 activity and upstream 5-HTP availability, through changes in 5-HT receptor signaling and SERT function, to downstream perturbations in the NAS–melatonin anabolic axis and the 5-HIAL–5-HIAA/5-HTOL catabolic balance. Each metabolic branch carries specific biological consequences for gonadal function, gamete quality, and re-productive resilience—and each represents a measurable endpoint for the integrative serotoninomic toxicological framework we propose [9,11].

### 3.2. Receptors, Transporters, and Neuroendocrine Crosstalk

5-HT interacts with seven distinct receptor families, 5-HT_1_, 5-HT_2_, 5-HT_3_, 5-HT_4_, 5-HT_5_, 5-HT_6_, and 5-HT_7_ each consisting of multiple subtypes that increase the functional diversity of serotonergic signalling. The 5-HT_1_ family includes subtypes 5-HT_1_A, 5-HT_1_B, 5-HT_1_D, 5-HT_1_E, and 5-HT_1_F, all coupled to Gi/Go proteins and mainly inhibitory. The 5-HT_2_ family comprises 5-HT_2_A, 5-HT_2_B, and 5-HT_2_C subtypes, which are coupled to Gq/G11 and mediate excitatory effects. The 5-HT_3_ receptor is the only ligand-gated ion channel in the family and is permeable to Na^+^, K^+^, and Ca^2+^. The 5-HT_4_, 5-HT_6_, and 5-HT_7_ receptors couple to Gs proteins and activate adenylyl cyclase, while 5-HT_5_ receptors include 5-HT_5_A and 5-HT_5_B subtypes, both coupled to Gi. These receptors are broadly distributed in the central and peripheral nervous systems. Notably, 5-HT_2_A and 5-HT_7_ are present in ovarian and testicular tissues, where they participate in follicular development, sperm motility, and hormonal feedback regulation [13,14].

The serotonin transporter (SERT), which mediates synaptic reuptake and clearance, is also present in the placenta and uterus, supporting its proposed role in embryonic development and maternal–foetal communication [18,32]. In reproductive biology, serotonergic signalling interacts with neuroendocrine axes, affecting gonadotropin release, steroid hormone production, and sexual behaviour.

Experimental evidence shows that serotonin influences the hypothalamic–pituitary–gonadal (HPG) axis by modulating the activity of Gonadotropin-Releasing Hormone-producing neurons, both directly and via other neurochemical pathways, including kisspeptin pathways, which are crucial regulators of the pulsatile release of Gonadotropin-Releasing Hormone. Serotonin also impacts the secretion of Luteinizing Hormone and Follicle-Stimulating Hormone, although these effects differ across hypothalamic regions, stages of the reproductive cycle, and overall hormonal status [33,34,35].

Disruption of serotonergic activity, whether caused by pharmacological agents or environmental toxins, can interfere with reproductive cycles, impair mood regulation, and reduce fertility [35,36]. This neuroendocrine communication is crucial to the serotoninomic model.

PERM, known for its neuroactive properties [6,10,37,38], may disrupt serotonergic balance by affecting receptor function, transporter activity, or intracellular signalling [39]. Several experimental studies have demonstrated that pyrethroid exposure decreases serotonin (5-HT) levels across key brain regions in rodent models [30]. It was shown that repeated intraperitoneal administration of type II pyrethroids deltamethrin (20 mg/kg/day), cyfluthrin (14 mg/kg/day), and lambda-cyhalothrin (8 mg/kg/day) for 6 days in adult male Wistar rats caused significant reductions in 5-HT and its metabolite 5-HIAA in the frontal cortex, hippocampus, midbrain, and striatum, with accelerated serotonin turnover in the midbrain and striatum [30]. Complementary evidence from oral exposure confirmed these effects: Rodríguez et al. showed that cyfluthrin administered orally at 5–20 mg/kg/day for 6 days to male rats induced dose- and brain region-dependent decreases in 5-HT and 5-HIAA across the hypothalamus, midbrain, hippocampus, striatum, and prefrontal cortex [40]. The functional consequences of pyrethroid-induced serotonergic disruption were further demonstrated by Hossain et al., who used in vivo microdialysis in conscious male Sprague–Dawley rats to show that acute deltamethrin exposure (10–60 mg/kg i.p.) dose-dependently decreased striatal 5-HT release to 58–32% of baseline, along with behavioural signs including aggressive behaviour and hyperactivity [3]. Critically, Nasuti et al. demonstrated that neonatal permethrin exposure at 34 mg/kg/day (oral gavage, PND6–21)—a dose close to the NOAEL that did not cause overt signs of toxicity—was enough to induce striatal neurodegeneration, evidenced by reductions in dopamine and Nurr1, increased lipid peroxidation, and imbalances in 5-HT and noradrenaline in the medial prefrontal cortex during late adulthood [37].

Understanding these mechanisms is essential for guiding future research and creating new models that explain how serotonergic disruption influences reproductive and behavioural outcomes [8]. Serotoninomics offers a comprehensive framework for studying these phenomena and devising strategies for mitigation and resilience [9].

## 4. Why Permethrins? Justification of Compound Selection and Relevance to Public Health

Permethrin (PERM) is a member of the synthetic class I pyrethroids. It is a cyclopropanecarboxylate ester, specifically derived from 3-(2,2-dichlorovinyl)-2,2-dimethylcyclopropanecarboxylic acid. It has been associated with whole-body tremors, aggressive behaviour, hypersensitivity, and ataxia [38,41]. Its mechanism of action involves changes in sodium channel conformation during their opening and closing in neuronal membranes [41,42]. It is widely used in agriculture and residential homes, found in household sprays, aerosols, insect repellents, pet shampoos, and lotions. It is also used directly on humans to treat dermal parasites such as scabies and pediculosis (lice) [38,41]. PERM has been shown to affect the reproductive, skeletal, cardiovascular, immune, and nervous systems, causing cardiotoxicity, endocrine dysfunction, hepatotoxicity, and cytotoxicity [38,43,44]. PERM acts on glia and neurons, decreases neurogenesis, and causes partial neuron loss and mild inflammation. It increases pro-inflammatory cytokines, mainly Tumor Necrosis Factor-alpha (TNF-α), and contributes to neuroinflammation that underlies several neurodegenerative diseases. Studies using in vitro and animal models indicate that pyrethroid exposure activates microglia and stimulates the release of proinflammatory cytokines, leading to chronic neuroinflammation [38,41,42,43,44,45].

Their popularity stems from a perceived safety profile: they are rapidly biodegradable, show low mammalian toxicity in acute-exposure models, and are approved by regulators for use in children and pregnant women [5,6,46]. This reputation has led to widespread, often unregulated exposure, especially in urban and peri-urban areas where reproductive-age populations are frequently exposed. However, this confidence is not fully supported by strong long-term safety data. From a serotoninomic perspective, PERM can act as a disruptive compound that interferes with steroidogenesis and crosses the blood–brain barrier, thereby affecting neurotransmitter activity, including serotonergic functions [5,6,46].

Prolonged or developmental exposure to permethrins has been associated with anxiety-like behaviours, altered sexual behaviours, and decreased fertility observed in different models [2,5,6,46]. Although PERM is lipophilic, current evidence suggests that its organ-specific effects result from the pathophysiological mechanisms it induces rather than from bioaccumulation [37]. Human studies following topical application show low systemic exposure, with nearly complete elimination within a week [47]. For example, applying 25 mg of PERM to the forearm for 8 h resulted in approximately 0.2% absorption, with baseline levels returning within 72 h and elimination via blood, saliva, or urine continuing for up to 7 days [48]. However, lipophilicity may still affect distribution and retention in fatty tissues, and long-term exposure has been linked to liver and kidney damage, raising concerns about cumulative effects. Notably, tissues such as the brain and gonads, which are rich in serotonergic receptors and SERT, could be more vulnerable to PERM’s effects than other organs. This is especially worrying given the compound’s presence in products marketed for maternal and pediatric use, including lice treatments, mosquito repellents, and household sprays [38,49]. These exposures raise ethical and epidemiological questions regarding vulnerability during gestation and early life.

Although PERM is not currently classified as a reproductive toxicant under conventional frameworks, emerging evidence suggests it may subtly disrupt neuroendocrine balance, especially through serotonergic pathways [1,2,50,51]. By using PERM as our model compound, we aim to challenge traditional toxicology and promote comprehensive risk assessments that consider emotional, behavioural, and neurochemical factors. PERM exemplifies the intersection of accessibility, invisibility, and neurochemical disruption. They are silent agents woven into everyday life. Through serotoninomics, we aim to make their impacts visible, measurable, and ethically manageable (Table 1).

## 5. Implications for Toxicological Modelling: Toward Integrative and Predictive Frameworks

Traditional toxicological models often focus on reductionist endpoints like hormone levels, organ weights, and histopathology, while ignoring the neurochemical and behavioural factors that influence reproductive resilience. This gap is especially clear when evaluating neuroactive substances such as PERM, which can bypass traditional endocrine markers but still significantly disrupt serotonergic signalling and emotional regulation [1,2,3,4,5,6].

Serotoninomics proposes a paradigm shift: moving from isolated biomarkers to comprehensive frameworks that include molecular, behavioural, and emotional outcomes. This involves incorporating receptor profiling, neurotransmitter quantification, and behavioural assays into toxicological protocols [3,8,44,50]. It also requires longitudinal designs that capture developmental windows of vulnerability, such as gestation, puberty, and reproductive maturity, during which serotonergic systems are particularly plastic and sensitive [48,51,55,57].

A defining characteristic of the Serotoninomic approach to toxicology—and one that fundamentally distinguishes it from conventional single-endpoint models—is its capacity to evaluate permethrin’s impact not on isolated serotonergic molecules but on the 5-HT grouped as an integrated biochemical system; this group encompasses the full indolamine cascade: from upstream precursor availability (L-tryptophan → 5-HTP), through the biosynthetic node (5-HTP → 5-HT via TPH1/TPH2 and AADC), along the catabolic branches (5-HT → 5-HIAL → 5-HIAA via ALDH; 5-HIAL → 5-HTOL via ALR/ADH), and through the anabolic axis (5-HT → NAS → melatonin via AANAT/HIOMT). Permethrin’s toxicological signature within this system is not a single disruption—it is a cascade perturbation that can simultaneously alter TPH1 activity and local 5-HT synthesis in testicular and epididymal tissues—where the serotonergic machinery has been fully characterized [7,15,62]—through mechanisms including disruption of serotonergic turnover documented after pyrethroid exposure [30], a shift in the ALDH/ALR balance toward 5-HTOL accumulation under permethrin-induced oxidative stress [19,30], suppression of the NAS–melatonin anabolic axis, and reduced 5-HT receptor-mediated signalling in Leydig cells, Sertoli cells, and spermatozoa—where these receptors are expressed and functionally active [7,9,15]—as part of a systemic cascade perturbation [9].

The toxicological significance of this cascade perspective is twofold: first, it predicts that PERM exposure will produce a pattern of metabolic disruption—measurable across multiple nodes simultaneously—rather than a single biomarker alteration; and second, it establishes that the reproductive consequences of serotonergic disruption by PERM are mechanistically linked not only to 5-HT depletion but also to the downstream loss of melatonin-dependent gonadoprotection [24], NAS-mediated neurotrophic support in germ cells [22], and the redox imbalance signalled by 5-HTOL accumulation in spermatogenic tissue [19,31]. This 5-HT group-based integrated toxicology, the systematic characterization of permethrin’s impact across every node of the serotonergic metabolic network, is the scientific core of the Serotoninomic framework and its most important contribution to reproductive toxicology [9,11].

Predictive modelling must progress to include neuroendocrine crosstalk, emotional phenotyping, and transgenerational effects. For example, gestational exposure to PERM might not cause immediate physical defects but could influence maternal behaviour, induce anxiety in offspring, and impair reproductive functions in adulthood [43,63,64]. It is also important to recognize that gestational exposure to permethrin can alter foetal development [64,65,66,67]. The detection of PERM metabolites, 3-phenoxybenzoic acid, and cis/trans isomers of dichlorocinnamic acid in umbilical cord blood confirms that PERM and its metabolites cross the placenta and reach the foetus, indicating they could affect development during pregnancy [67]. This evidence shows that PERM can impact the foetus during critical neurodevelopmental stages.

Epidemiological and experimental research shows that prenatal exposure to pyrethroids is associated with changes in neuronal development, including disruptions of voltage-gated sodium channels, oxidative stress, and alterations in gene expression related to neurogenesis [5,66,67]. Several studies have reported a higher risk of low birth weight, smaller head circumference, and delays in motor and cognitive development during early childhood among children prenatally exposed to PERM. Follow-up studies of this cohort have identified changes in children’s adaptive behaviour and executive functions. These outcomes vary with the dose and duration of exposure [67,68,69]. Therefore, the evidence suggests that PERM, even at levels considered safe, can have subtle yet lasting effects on neurological health and child development. Such effects often remain undetected by conventional toxicology but are important concerns for public health, reproductive health justice, and overall health and well-being [32,51,65,66,67].

Serotoninomic modelling encourages the integration of transdisciplinary data from molecular biology, behavioural neuroscience, and environmental epidemiology to create risk profiles that reflect real-world complexity. It is no longer sufficient to measure hormone levels alone.

We need to examine how this compound influences bonding, desire, resilience, and reproductive agency. By incorporating serotonergic pathways into toxicological models, we advance toward a science that is not only predictive but also compassionate, capable of understanding the emotional and relational aspects of reproductive health and responding with epistemological tenderness and ethical accuracy.

### 5.1. Epidemiological Evidence and Public Health Impact

Epidemiological studies increasingly indicate that exposure to pyrethroids like permethrin is not insignificant, especially for vulnerable groups such as pregnant women and children. In Mexico and Latin America, pesticide exposure during pregnancy has been linked to intrauterine growth restriction and developmental delays after birth [70,71]. International reviews covering more than 200 studies highlight harmful effects on female reproductive health, including ovarian issues and hormonal imbalances [69,72]. Prenatal exposure to permethrin has been associated with lower birth weight, smaller head circumference, and challenges with motor and cognitive skills in early childhood [68,69]. These findings raise serious public health concerns, particularly given how common and often unregulated permethrin use is in homes, agriculture, and medical products targeted at maternal and pediatric care [50]. Agencies such as the U.S. EPA recognize the limitations of current toxicology models, which frequently fail to account for neurochemical and behavioural effects [73]. Unlike well-known endocrine disruptors such as bisphenol A, permethrin remains largely overlooked in public health discussions, despite evidence of its subtle yet persistent effects on serotonin pathways and reproductive health [74,75].

### 5.2. Comparative Analysis with Other Neuroactive Toxicants

Permethrin is a neuroactive compound whose subtle yet lasting effects often go unnoticed in traditional toxicology tests. However, it is not the only one. Other environmental toxicants, such as bisphenol A (BPA) and phthalates, have also been shown to disrupt serotonergic and neuroendocrine pathways through different mechanisms. BPA, which is commonly found in plastics and food packaging, mainly binds to estrogenic receptors but has increasingly been linked to neurological problems, including changes in cognition, mood regulation, and synaptic health [74,75]. Phthalates, often used as plasticizers, interfere with steroid hormone production and have been connected to depressive behaviours and serotonergic imbalance in both animal studies and humans [74]. New evidence indicates that these compounds, like permethrin, can affect more than just endocrine systems—they also influence neurotransmitter signalling and neurodevelopmental processes [74].

From a serotoninomic perspective, the comparison highlights a critical gap: while BPA and phthalates have garnered public attention and regulatory focus, permethrin remains mostly overlooked despite evidence of its neurochemical and behavioural effects. This disparity emphasizes the need for comprehensive toxicological frameworks that assess neuroactive compounds not only for hormonal effects but also for their potential to disrupt serotonergic circuits, emotional resilience, and reproductive health.

### 5.3. Proposed Biomarkers for Serotoninomic Toxicology

A key step toward establishing serotoninomics as a framework for reproductive toxicology is identifying reliable biomarkers that capture neurochemical disruption beyond traditional endocrine endpoints.

The Serotoninomic approach to biomarker development departs fundamentally from the single-molecule paradigm of conventional toxicology. Rather than proposing isolated indicators, it frames PERM-induced serotonergic disruption as a cascade signature—a coordinated pattern of alterations across the full 5-HT metabolic group that must be captured simultaneously to reflect the true biological impact of exposure. This panel-based logic encompasses six interconnected biomarker nodes: (i) upstream biosynthetic capacity, indexed by TPH1 expression and 5-HTP availability in gonadal tissues [7]; (ii) serotonin itself (5-HT) and its turnover ratio in seminal plasma and testicular tissue [15]; (iii) the catabolic oxidative endpoint, 5-HIAA, as an indicator of MAO activity and serotonergic flux [30]; (iv) the catabolic reductive endpoint, 5-HTOL, as a redox-sensitive biomarker reflecting mitochondrial dysfunction and NADH/NAD^+^ im-balance in spermatogenic cells [19,31]; (v) N-acetylserotonin (NAS), whose circadian suppression signals disruption of TrkB-mediated neurotrophic support in germ cells [21,22]; and (vi) melatonin and its gonadal receptor expression profile (MT1/MT2), as the terminal indolamine biomarker of anabolic serotonergic integrity in Leydig cells, Sertoli cells, and oocytes [24,25]. Together, this six-node panel constitutes the first operationalized Serotoninomic Biomarker Panel for PERM-induced reproductive toxicology, transforming the 5-HT group from a series of isolated analytes into a coherent, mechanistically grounded toxicological fingerprint.

Several candidates have emerged from both experimental and clinical studies. First, serotonin metabolites such as 5-hydroxyindoleacetic acid (5-HIAA) serve as measurable indicators of serotonergic turnover and have been shown to increase in specific brain regions following permethrin exposure [52,60]. Second, the serotonin transporter (SERT), found in placental and gonadal tissues, is a crucial biomarker of maternal–foetal communication and reproductive vulnerability, with altered expression linked to impaired fertility and developmental outcomes [76]. Third, receptor profiling of 5-HT_2_A and 5-HT_7_ receptors in ovarian and testicular tissues provides insight into how permethrin affects follicular development, sperm motility, and hormonal feedback loops [15].

Beyond molecular markers, integrative approaches suggest combining biochemical assays with behavioural phenotyping. For example, linking changes in serotonergic metabolites to anxiety-like behaviours or reproductive issues in animal models provides a multidimensional biomarker strategy [76,77]. Epigenetic modifications in genes that regulate serotonin production and receptor expression, particularly in gametes, are also emerging as potential transgenerational biomarkers of exposure [77,78,79]. Finally, advances in neuroimaging, such as positron emission tomography (PET), enable in vivo visualization of serotonergic activity, providing translational biomarkers that connect animal and human studies [80,81,82].

This integrated serotoninomic panel, covering biosynthesis, catabolism, receptor, and transporter aspects of the 5-HT system, shifts reproductive toxicology from a single endpoint to a systems-level assessment of PERM-induced serotonergic disruption [9,11].

Together, these biomarkers can enhance toxicological models by incorporating serotonergic pathways into risk assessment, thereby improving predictive accuracy and relevance to public health.

### 5.4. Policy and Regulatory Perspectives

Despite widespread use of permethrin in agriculture, households, and medical products, regulatory frameworks continue to classify pyrethroids as relatively safe, mainly focusing on acute toxicity and traditional endocrine effects. The U.S. Environmental Protection Agency (EPA) has carried out registration reviews and ecological risk assessments for pyrethroids, but recent white papers highlight the inadequacy of current physiologically based pharmacokinetic (PBPK) models in accounting for uncertainty factors in risk assessment [83]. EPA human health risk assessments address aggregate exposure but often overlook neurochemical and behavioural outcomes relevant to serotoninomics.

Internationally, the Agency for Toxic Substances and Disease Registry (ATSDR) has established minimal risk levels (MRLs) for oral exposure to permethrin but admits there is insufficient data for inhalation and chronic exposure routes [84]. This regulatory gap is especially troubling given the widespread use of the compound in pediatric and maternal care products, where long-term exposure might subtly affect serotonergic pathways. Compared with endocrine disruptors like bisphenol A, which have led to bans and public health campaigns, permethrin remains largely overlooked in regulatory discussions. This gap highlights the need for updated toxicological models that incorporate serotonergic biomarkers, behavioural endpoints, and transgenerational effects. Policy frameworks must evolve to recognize neuroactive compounds as reproductive toxicants, ensuring that precautionary principles guide their use and disposal.

### 5.5. Future Research Directions

Future research must address critical gaps in understanding how permethrin and related pyrethroids disrupt serotonergic systems across different developmental stages and generations. Long-term mammalian studies are necessary to evaluate early-life exposure and track effects into adulthood, focusing on impacts on fertility, cognition, and emotional regulation [85]. Human cohort studies should measure permethrin metabolites in biological fluids and link them to serotonergic biomarkers, mental health indicators like anxiety and depression, and cognitive performance [86]. Techniques such as positron emission tomography (PET) can provide translational insights into serotonergic activity following exposure, linking animal models to human studies [81,82].

Epigenetic analyses of genes involved in serotonin synthesis, transport, and receptor expression represent another frontier, offering potential markers of transgenerational vulnerability [79]. Including sex-based analyses is also essential, as evidence indicates different serotonergic responses between males and females [87].

Finally, computational approaches such as machine learning and systems biology models can integrate molecular, behavioural, and epidemiological data, creating predictive frameworks that capture real-world complexity [87,88]. These approaches will advance serotoninomic toxicology, helping risk assessments to incorporate neurochemical, behavioural, and transgenerational factors.

## 6. Call for Transdisciplinary Exploration: Bridging Science, Policy, and Clinical Awareness

The serotoninomic approach to reproductive toxicology is more than just a scientific idea; it is a call to action. It urges researchers, clinicians, policymakers, and educators to see serotonin as a vital factor in reproductive health, emotional resilience, and environmental vulnerability. Traditional toxicology has long operated within disciplinary silos, separating molecular data from behavioural outcomes and excluding emotional and relational dimensions from risk assessment. It’s omic science challenges this fragmentation, offering an integrative framework that bridges neurochemistry, reproductive biology, and public health [89].

Clinicians must recognize that compounds such as PERM, although not classified as endocrine disruptors, may influence fertility, mood, and bonding through serotonergic pathways. Policymakers should consider updating exposure guidelines to include neurochemical effects and behavioural indicators. Educators and researchers need to collaborate to develop new methods that reflect the complexity of real-world exposure, such as receptor mapping, emotional phenotyping, and transgenerational studies [56,61,65,89]. This transdisciplinary approach is not just theoretical; it is urgent.

In a world where insecticides are sprayed in nurseries, where emotional disorders are increasing alongside environmental degradation, and where invisible molecules affect reproductive health, we must consider what kind of science can defend life, desire, and human wholeness. It provides one comprehensive answer. It is a science of bridges linking molecules and memories, exposure and emotion, data and care. It offers not only new biomarkers but also new responsibilities.

**Figure 1:**
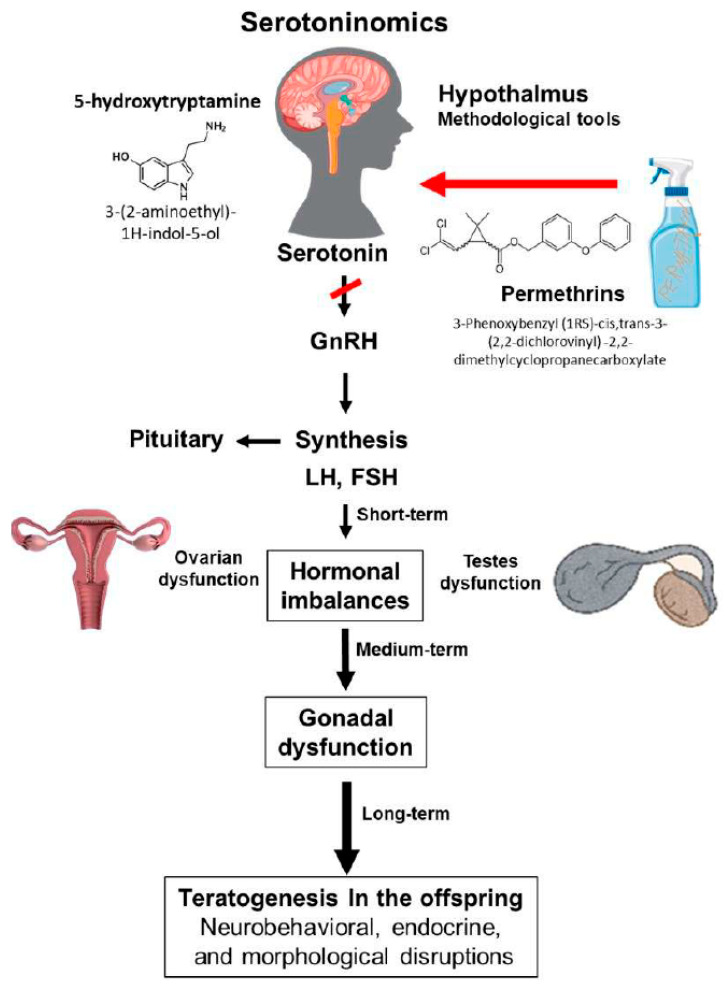
Representation of the biochemical pathway affected by permethrin exposure.

From a serotonergic perspective, various findings suggest that PERM exposure disrupts hormonal balance in adults and may cause teratogenesis in sperm, damaging the epigenetic and functional integrity of male gametes. These effects are significant and could lead to teratogenesis in offspring, including neurobehavioral, endocrine, or morphological issues [1,2,48,50]. The interference of PERM with the serotonergic-HPG axis during critical developmental periods highlights the need to evaluate these compounds not just as neurotoxic agents but also as transgenerational disruptors [30,39]. This involves updating reproductive toxicity models by adding serotonergic biomarkers, conducting epigenetic analyses in gametes, and performing long-term studies of offspring (Figure 1).

## 7. Challenges in Examining Permethrin’s Effect on Serotonergic Systems

According to serotoninomics, several findings have emerged that highlight the complexity of studying permethrin’s effects on serotonergic systems.

(1) Changes in serotonin breakdown products after acute exposure. In rats treated with permethrin, 5-HT levels remain largely stable, whereas levels of its main metabolite, 5-HIAA, increase in brain regions including the hypothalamus, brainstem, hippocampus, and striatum [1].
(2) Developmental exposure and lasting effects. In young rats exposed to relatively low doses of permethrin before and after birth, researchers observed impairments in learning and memory, alterations in immature neurons, changes in astrocyte morphology, and disruptions in neural circuitry [1,3,5,46,50,90].
(3) Neurochemical imbalances extend beyond serotonin. Early research indicates that dopamine and norepinephrine, in addition to serotonin, may also be affected, with region-specific imbalances contributing to behavioural and cognitive changes [1,3,30,39].
(4) Behavioural changes associated with neuroinflammation. In animal models of Gulf War Illness, co-exposure to permethrin and stress led to depression-like behaviours, activated microglia, disrupted synaptic function, and altered neurotransmitter receptor regulation. Although 5-HT was not always measured directly, the term “chemical synaptic transmission” often implicitly included serotonergic involvement [38,44,46].
(5) Indirect mechanisms of disruption include oxidative stress (such as mitochondrial dysfunction), neuroinflammation (like elevated cytokines), and synaptic damage (alterations in density and morphology), which may indirectly impair serotonergic circuits [38,46,69,70].
(6) Dose, developmental timing, and regional specificity. Different or repeated doses of permethrin produce more pronounced effects. Developing brains are especially vulnerable, and different brain regions exhibit varying susceptibilities [38,68,69].
(7) Incomplete mapping of serotonin pathways. Although evidence indicates functional disturbances in 5-HT synthesis (e.g., through tryptophan hydroxylase), receptor interactions (e.g., 5-HT_1_, 5-HT_2_), reuptake (via SERT), degradation (via monoamine oxidase), and synaptic release, the data remain fragmented and inconclusive [3,10,11,13].

Beyond PERM, other silent disruptors, such as phthalates, bisphenol A (BPA), and certain organophosphates, may also interfere with serotonergic systems through indirect mechanisms. These compounds, commonly found in plastics, pesticides, and industrial waste, have been linked to oxidative stress, neuroinflammation, and hormonal imbalances, all of which can affect serotonin production, receptor expression, and synaptic health. For example, changes in serotonergic pathways caused by phthalates have been associated with depressive behaviours [74,75]. Meanwhile, the neuroimmune toxicity of BPA and its analogues, including potential effects on 5-HT-related circuits, has been highlighted [91].

Despite their widespread presence and low-dose exposure, the long-term effects of these silent disruptors on emotional regulation, cognitive development, and reproductive health remain not fully understood. Serotoninomics provides a framework for studying these compounds as both toxic agents and as factors influencing vulnerability and resilience across generations.

## 8. Critical Gaps in Serotoninomic Research on Permethrin

Currently, there are critical gaps in serotonin research on permethrin, including the following:

(1) Limited research specifically on 5-HT. Most studies broadly examine monoamines or emphasize dopamine and behavioural outcomes, without fully exploring serotonergic receptors, transporters, synthesis, and regulation [9].
(2) Heterogeneity in experimental models. Variations in species (rats, mice, fish), dosing regimens, routes of administration (oral, dermal), and developmental stages (prenatal, neonatal, adult) complicate cross-study comparisons [16,38,83].
(3) Lack of dose relevance. Many animal studies use high or non-comparable doses, which limits their applicability to real-world human exposures [38,67,67].
(4) Influence of interacting variables. Stress, co-exposure to pollutants, diet, immune status, age, and sex all influence neurotoxic outcomes, especially in serotonergic systems [1,3,6,50].
(5) Distinction between acute and chronic exposure. Understanding adaptation and cumulative effects over time remains a critical gap [49,91,92].
(6) Temporal limitations. Most studies assess short-term outcomes; few examine long-term or intergenerational effects, particularly in mammals [38,93].
(7) Disconnect between biochemical and functional outcomes. Measuring serotonin levels or receptor activity is insufficient without linking them to behavioural or cognitive endpoints [6,17].
(8) Regional and cellular specificity. Serotonin’s role differs across brain regions and cell types. Studies must include glial cells (astrocytes, microglia) to fully understand synaptic- and network-level disruptions [92].
(9) Need for pharmacokinetic data. Understanding how permethrin and its metabolites distribute, accumulate, and persist in the brain is crucial [48,92].
(10) Human relevance and safety. More environmental and occupational research is needed to assess behavioural and serotonergic outcomes in exposed populations, thereby distinguishing direct serotonergic effects from those mediated by inflammation, oxidative stress, or mitochondrial dysfunction [92,93].

## 9. Conclusions

### Toward a Resonant Science of Serotoninomics

Serotonergic medicine does not aim to replace toxicology but to supplement it. By including emotional, behavioural, and transgenerational factors, it promotes a comprehensive science capable of protecting human life and the relationships that support it. Our goal is clear: to position serotonergic medicine within the broader omics landscape, alongside genomics, proteomics, and metabolomics, as a transdisciplinary field spanning molecular, cellular, clinical, and environmental levels. This encompasses public health, clinical practice, and policymaking.

Confronted with silent disruptors like PERM, we need to ask not only what is toxic but also what is invisible, as lasting effects remain in exposed organisms and their environments. The use and disposal of these compounds must be carefully evaluated, regulated, and guided by precautionary principles that focus on long-term safety. An area still requiring research is the potential impact of PERM exposure on central nervous system disorders such as Alzheimer’s, Parkinson’s, schizophrenia, and dementia, especially those involving serotonin receptor 5-HT_2_A. This receptor is essential for cognition, perception, and emotional regulation, and its disruption may contribute to neuropsychiatric and neurodegenerative diseases. Studying how environmental toxins interact with serotonergic circuits in these disorders could provide new insights into vulnerability and resilience in brain health.

Serotoninomics provides alternative insights. It is a science that links molecules to memory, emotion, and resilience, and it does more than just offer a new perspective on toxicology—it calls for a paradigm shift impacting science, medicine, and society. By incorporating serotonergic biomarkers into reproductive toxicology, we can move beyond simple endpoints and adopt a holistic approach that protects not only biological functions but also emotional resilience and intergenerational health. This vision encourages clinicians to notice subtle neurochemical disruptions, policymakers to include serotonergic pathways in regulations, and researchers to conduct long-term, interdisciplinary studies exploring the full spectrum of vulnerabilities. In this way, omics science positions itself as a meaningful field capable of connecting molecules to memory, emotional exposure, and data to care. This resonance gives reviewers and readers a compelling reason to recognize serotoninomics as a vital addition to toxicology, ensuring that hidden disruptors like permethrin are identified both scientifically and through public health efforts. It is now ready to be applied with accuracy, compassion, and responsibility.

## 10. Future Directions

Based on what was previously described, the following should be considered:

(1) Investigate how PERM affects specific serotonergic receptors (e.g., 5-HT_1_A, 5-HT_2_A/C) in different brain regions, including receptor regulation.
(2) Examine SERT to evaluate changes in expression or activity during development and adulthood.
(3) Conduct long-term mammalian studies on early-life exposure, including follow-up into adulthood to identify lasting effects.
(4) Initiate human cohort studies that measure PERM metabolites and examine their correlation with 5-HT levels, mental health indicators, and cognitive performance.
(5) Use functional imaging, such as Positron Emission Tomography, in animal and human models to visualize serotonergic activity after exposure.
(6) Explore the combined effects of PERM and stress on serotonergic pathways.
(7) Incorporate sex-based analyses, acknowledging distinct serotonergic responses in males and females.
(8) Investigate epigenetic changes in genes related to 5-HT synthesis, receptors, and signalling after early exposure.

## Figures and Tables

**Table 1:** Classical endocrine disruptors vs. neuroactive compounds.

Feature	Classical Endocrine Disruptors (e.g., Bisphenol A (BPA), Phthalates, Parabens, Pesticides)	Neuroactive Compounds (e.g., PERM)	Health Damage Examples:
Primary target	Steroid hormone synthesis and receptor binding	Neurotransmitter signalling (e.g., serotonin, dopamine)	Serotonin (5-HT): Regulates mood, sleep, appetite, and cognition. Dopamine (DA): Controls reward, motivation, motor function, and learning. Low serotonin → Depression, anxiety, insomnia. High dopamine → Psychosis, schizophrenia. Low dopamine → Parkinson’s disease (motor dysfunction). Imbalance of → bipolar disorder, addiction, compulsive behaviours [52].
Mechanism of action	Estrogenic or anti-androgenic activity	Modulation of ion channels and neurochemical balance	Estrogenic activity (agonist). Modulates calcium and potassium channels; it enhances synaptic plasticity. Increased risk of hormone-dependent cancers (e.g., breast cancer) [53]. Anti-androgenic activity, Alters GABAergic and dopaminergic signalling; disrupts excitatory/inhibitory balance. Male infertility, reduced sperm quality [54].
Affected systems	Reproductive organs, endocrine glands	Central nervous system, reproductive tissues, and emotional regulation	Male fertility: Reduced sperm quality and motility due to endocrine disruption [51]. Female fertility: Altered ovarian reserve and disruption of the hypothalamic–pituitary–ovarian axis [55]. Embryonic development: Potential risks during pregnancy, affecting foetal growth and reproductive tissue formation [56].
Behavioural impact	Altered sexual development and fertility	Anxiety-like behaviour, impaired mating, and emotional dysregulation	Occupational exposure: Farmers exposed to mixed pesticide use show higher infertility rates and an increased risk of neurobehavioral disorders. Prenatal exposure: Children exposed in utero may experience developmental delays, altered sexual maturation, and emotional instability. Animal models: Rats exposed to organophosphates and pyrethroids exhibit anxiety-like behaviour, reduced fertility, and impaired mating patterns [51,57,58].
Exposure context	Plastics, cosmetics, food packaging	Insecticides, repellents, and household sprays	Occupational pesticide exposure links to infertility, depression, and hormone-related cancers. Cosmetic chemicals cause early puberty and menstrual issues. Rodents exposed to phthalates have reduced testosterone and mating problems. Zebrafish exposed to permethrin show anxiety-like behaviour and reproductive effects across generations toxicity [51,55].
Regulatory status	Often classified as endocrine disruptors	Generally considered safe; not classified as reproductive toxicants	Phytoestrogens such as soy isoflavones and flaxseed lignans bind to estrogen receptors and may benefit cardiovascular and bone health without adverse effects at dietary levels. Caffeine and certain plant compounds can influence hormonal pathways but are not harmful endocrine disruptors. Some regulated food additives are deemed safe at permitted levels by agencies such as EFSA and the FDA [59].
Serotoninomic relevance	Indirect modulation via hormonal pathways	Direct interference with serotonergic signalling	Indirect modulation includes sex hormones, cortisol, and endocannabinoids, which affect serotonin and neuronal excitability. Estrogens boost serotonin; cortisol degrades it via MAO-A, thereby reducing brain levels; endocannabinoids help regulate stress. Direct interference by SSRIs with SERT prolongs serotonin in the synaptic cleft [60].
Risk assessment gaps	Focused on hormonal endpoints	Neglects neurochemical and behavioural dimensions	Neurochemical: Relying only on cortisol levels can miss its role in depleting hippocampal serotonin. Neuronal Plasticity: Hormonal tests don’t measure BDNF synthesis, which is vital for brain repair; normal hormone levels don’t ensure this. Behavioural: Focusing solely on the adrenal gland ignores how a person manages frustration or fear. Changes in dopamine and serotonin can worsen impulsivity and aggression before organic issues are noticeable [61].

## Data Availability

The original contributions presented in this study are included in the article. Further inquiries can be directed to the corresponding author.

## Abbreviations

PERM	permethrin (s)
5-HT	serotonin
SERT	serotonin transporter
HPG	hypothalamic–pituitary–gonadal BPA: bisphenol A
5-HIAL	5-hydroxyindoleacetaldehyde
5-HTOL	5-hydroxytryptophol
NAS	N-acetylserotonin
AANAT	arylalkylamine N-acetyltransferase
HIOMT/ASMT	hydroxyindole-O-methyltransferase/acetylserotonin O-methyltransferase
ALR	aldehyde reductase
TrkB	tropomyosin receptor kinase B
BDNF	brain-derived neurotrophic factor
MT1/MT2	melatonin receptor type 1/melatonin receptor type 2
NADH/NAD^+^	nicotinamide adenine dinucleotide (reduced/oxidized forms)
AADC	aromatic L-amino acid decarboxylase
